# Bayesian Technique for the Selection of Probability Distributions for Frequency Analyses of Hydrometeorological Extremes

**DOI:** 10.3390/e20020117

**Published:** 2018-02-11

**Authors:** Lu Chen, Vijay P. Singh, Kangdi Huang

**Affiliations:** 1 College of Hydropower & Information Engineering, Huazhong University of Science & Technology, Wuhan 430074, China; 2 Department of Biological and Agricultural Engineering & Zachry Department of Civil Engineering, Texas A&M University, College Station, TX 77843-2117, USA

**Keywords:** entropy theory, frequency analysis, hydrometeorological extremes, Bayesian technique, rainfall

## Abstract

Frequency analysis of hydrometeorological extremes plays an important role in the design of hydraulic structures. A multitude of distributions have been employed for hydrological frequency analysis, and more than one distribution is often found to be adequate for frequency analysis. The current method for selecting the best fitted distributions are not so objective. Using different kinds of constraints, entropy theory was employed in this study to derive five generalized distributions for frequency analysis. These distributions are the generalized gamma (GG) distribution, generalized beta distribution of the second kind (GB2), Halphen type A distribution (Hal-A), Halphen type B distribution (Hal-B), and Halphen type inverse B (Hal-IB) distribution. The Bayesian technique was employed to objectively select the optimal distribution. The method of selection was tested using simulation as well as using extreme daily and hourly rainfall data from the Mississippi. The results showed that the Bayesian technique was able to select the best fitted distribution, thus providing a new way for model selection for frequency analysis of hydrometeorological extremes.

## 1. Introduction

Frequency analysis of hydrometeorological extremes plays an important role in the design of structures, such as dams, bridges, culverts, levees, highways, sewage disposal plants, waterworks, and industrial buildings [1,2,3,4,5]. From a frequency analysis, the probability of an extreme event can be estimated, and the value of a T-year design event (e.g., rainfall or flood) can be calculated. One of the objectives of frequency analysis of hydrometeorological extremes therefore is to establish a relationship between a flood or rainfall magnitude and its recurrence interval or return period.

A multitude of distributions have been employed for frequency analysis of hydrometeorological extremes. For example, the Pearson Type three (P-III) distribution is recommended in China; the Log-Pearson type three (LPT 3) is used in the U.S and Australia; and generalized extreme value (GEV) distribution is usually employed in Europe. Frequency analysis of hydrometeorological extremes at a given site or location is usually performed based on an appropriate probability distribution, which is selected on the basis of statistical tests for extreme hydrometeorological data [6]. However, no single distribution has gained global acceptance [7,8]. The traditional method is to try a variety of distributions and choose the best fitted distribution based on a particular mathematical norm, such as a least square error or a likelihood norm [9]. The disadvantages of this method of choosing are that it is laborious because too many different distributions need to be tried and empirical choices of candidate distributions make the results subjective [9,10,11]. In order to overcome these disadvantages, the generalized distributions have recently gained a lot of attention because they have been shown to be an effective tool for frequency analysis of hydrometeorological extremes. The greatest advantage of these generalized distributions is that they provide sufficient flexibility to fit a large variety of data sets, which facilitates the selection and comparison of different distributions. For example, Papalexiou and Koutsoyiannis [9] concluded that the generalized beta distribution of the second kind (GB2), which includes commonly used exponential, Weibull, and gamma distributions as special cases, was a suitable model for rainfall frequency analysis because of its ability to describe both J-shaped and bell-shaped data. Chen et al. [10] and Chen and Singh [11] also used the generalized gamma (GG) and GB2 distributions for hydrological frequency analysis, respectively. The results demonstrated that these two distributions could fit hydrometeorological data well. The generalized distributions can be derived using entropy theory by specifying appropriate constraints. The theory also provides a way for efficient parameter estimation [12].

Selection of the most appropriate distribution is of fundamental importance in hydrometeorological frequency analysis, since a wrong choice could lead to significant error and bias in design flood or rainfall estimates, particularly for higher return periods, leading to either under- or over-estimation, which may have serious implications in practice [13].

A distribution is often selected on the basis of statistical tests or by graphical methods [14]. Selection criteria based on the Akaike Information Criterion (AIC), the Bayesian Information Criterion (BIC), and the Anderson–Darling Criterion (ADC) are widely used in hydrology [4,15]. Laio et al. [16] presented an objective model selection criterion based on the AIC, the Bayesian Information Criterion (BIC), and the Anderson–Darling Criterion (ADC). Using a rigorous numerical framework, they found that the ability of these criteria to recognize the correct parent distribution from the available data varied from case to case, and these were more effective for two parameter distributions [13]. In this study, a more objective method based on a Bayesian technique is introduced to select the distribution with more parameters for frequency analysis of hydrometeorological extremes.

Bayesian method has been widely used for hydrological analysis, such as model selection and hydrological uncertainty analysis. Duan et al. [17] used Bayesian model averaging for multi-model ensemble hydrologic prediction. Hsu et al. [18] used a sequential Bayesian approach for hydrologic model selection and prediction. Najafi et al. [19] used Bayesian Model Averaging method to assess the uncertainties of hydrologic model selection. Robertson and Wang [20] introduced a predictor selection method for the Bayesian joint probability modeling approach to seasonal streamflow forecasting at multiple sites. In addition, Bayesian model method was also used for model uncertainty analysis [21,22].

The objective of this study is therefore to present a more objective method based on a Bayesian technique to select the most appropriate generalized distribution for frequency analysis of hydrometeorological extremes. The entropy theory was employed to derive generalized distributions for hydrometeorological extremes and estimate their parameters based on the principle of maximum entropy. A simulation test was carried out to evaluate the performance of the proposed Bayesian model selection technique. The proposed method was then tested using annual maximum hourly and daily precipitation data from Mississippi.

## 2. Entropy Theory

Since the entropy theory was used for the derivation of these generalized distributions and estimation of their parameters, in this section, the entropy theory combined with the principle of maximum entropy (POME) method is introduced.

The entropy, defined by Shannon in 1848, can be expressed by

(1) $$H(X)=-\int_{0}^{\infty }f(x)\log f(x)dx$$

where *f*(*x*) is the probability density function (PDF) of *X. f*(*x*) can be derived by maximizing the entropy subject to given constraints, which can be expressed by

(2a) max H(X)

(2b) $$s.t.\;\int_{0}^{\infty }f(x)dx=1;$$

(2c) $$\int_{0}^{\infty }g_{i}(x)f(x)dx=C_{i}\;(i=1,\;\ldots ,\;m)$$

Employing the method of Lagrange multipliers, the PDF of *X* from Equations (1) and (2) can be derived as

(3) $$f(x)=\exp(-\lambda_{0}-\lambda_{1}g_{1}(x)-\lambda_{2}g_{2}(x)-\ldots -\lambda_{m}g_{m}(x))$$

where *m* is the number of constraints; and *λ_0_*, …, *λ_m_* are the Lagrange multipliers. According to (2b), *λ*_0_ can be defined as

(4) $$\exp(\lambda_{0})=\int_{0}^{\infty }\exp(-\lambda_{1}g_{1}(x)-\lambda_{2}g_{2}(x)-\ldots -\lambda_{m}g_{m}(x))dx.$$

When different constraints are used, different PDFs can be obtained. According to the POME theory, all of the generalized distributions discussed in the following can be written in the form of Equation (3).

## 3. Generalized Distributions

Five generalized distributions, namely the GG distribution, the GB2 distribution, and three Halphen family distributions, were used in this study. The principle of maximum entropy (POME) method was used for parameter estimation, and it involves the following steps: (1) specification of constraints and maximization of entropy using the method of Lagrange multipliers; (2) derivation of the relation between Lagrange multipliers and constraints; (3) derivation of the relation between Lagrange multipliers and distribution parameters; and (4) derivation of the relation between distribution parameters and constraints. Detailed information on obtaining the equations for parameter estimation of those generalized distributions is given in [10,11,23]. In this paper, we mainly focus on model selection based on the Bayesian method.

### 3.1. Generalized Gamma Distribution

The probability density function of the GG distribution is given by

(5a) $$f(x)=\frac{r_{2}}{\beta \Gamma (\frac{r_{1}}{r_{2}})}{\left(\frac{x}{\beta }\right)}^{\left(r_{1}-1\right)}\exp(-{\left(\frac{x}{\beta }\right)}^{r_{2}})$$

where *Γ*(·) is the gamma function; *r*_1_ and *r*_2_ are the shape parameters, *r*_1_ > 0, *r*_2_ > 0; and beta is the scale parameter, *β* > 0.

For deriving Equation (5a) from the entropy theory, the following constraints are specified:

(5b) $$\int_{0}^{\infty }f(x)dx=1$$

(5c) $$\int_{0}^{\infty }f(x)\ln xdx=E(\ln X)$$

(5d) $$\int_{0}^{\infty }f(x)x^{q}dx=E(X^{q}).$$

The probability density function (PDF) of the GG distribution can then be expressed as [10]:

(6) $$f(x)=\exp(-\lambda_{0}-\lambda_{1}\ln(x)-\lambda_{2}x^{q})$$

where *λ*_0_, *λ*_1_, and *λ*_2_ are the Lagrange multipliers, and *q* is the parameter $q=r_{2}$ [10].

The relations between Lagrange multipliers and parameters can be summarized as

(7) $$\left\{ \begin{array}{l} q=r_{2}\\ \lambda_{1}=1-r_{1}\\ \lambda_{2}=\beta^{-r_{2}} \end{array}\right..$$

The equations for parameter estimation based on the POME method can be given as [10]

(8) $$\left\{ \begin{array}{l} \frac{1}{r_{2}}\ln(\beta^{-r_{2}})-\frac{1}{r_{2}}\varphi (\frac{r_{1}}{r_{2}})=-E(\ln X)\\ \beta^{r_{2}}\frac{r_{1}}{r_{2}}=-E(\ln(X^{r_{2}}))\\ \frac{1}{{r_{2}}^{2}}\varphi^{\prime }(\frac{r_{1}}{r_{2}})=\mathrm{var}(\ln X) \end{array}\right.$$

where *φ* (·) is the digamma function; and *φ*’(·) is the tri-gamma function.

As seen in Equation (8), there are three unknown parameters, *r*_1_, *r*_2_, and β, in the three equations, and the variable *X* represents the observed hydrometeorological extreme series, which have been known before. By solving this equation set, the parameter of the GG distribution can be determined. The estimation procedures for other distributions are the same as those for the GG distribution.

### 3.2. Generalized Beta Distribution of the Second Kind

The PDF of the GB2 distribution is given by

(9a) $$f(x)=\frac{r_{3}}{bB(r_{1},r_{2})}{\left(\frac{x}{b}\right)}^{r_{1}r_{3}-1}{\left(1+{\left(\frac{x}{b}\right)}^{r_{3}}\right)}^{-(r_{2}+r_{1})}$$

where *B*(·) is the beta function; and *r*_1_, *r*_2_, and *r*_3_ are the shape parameters, *r*_1_ > 0, *r*_2_ > 0 and *r*_3_ > 0; and *b* is the scale parameter, *b* > 0.

For deriving Equation (9a) from the entropy theory, the following constraints are specified:

(9b) $$\int_{0}^{\infty }f(x)dx=1$$

(9c) $$\int_{0}^{\infty }f(x)\ln xdx=E(\ln X)$$

(9d) $$\int_{0}^{\infty }f(x)\ln{\left(1+px^{q}\right)}^{1/p}dx=E(\ln{\left(1+pX^{q}\right)}^{1/p}).$$

According the maximum entropy theory, the PDF of the GB2 distribution can be expressed as [11]

(10) $$f(x)=\exp(-\lambda_{0}-\lambda_{1}\ln(x)-\lambda_{2}\ln{\left(1+px^{q}\right)}^{1/p})$$

where *p* and *q* are two parameters, which are also related to the parameters of the GB2 distribution, $p={\left(\frac{1}{\beta }\right)}^{r_{3}}$, and $q=r_{3}$.

The relations between Lagrange multipliers and parameters can be summarized as

(11) $$\left\{ \begin{array}{l} \lambda_{1}=1-r_{1}q\\ \lambda_{2}=p(r_{2}+\frac{1-\lambda_{1}}{q})\\ p={\left(\frac{1}{\beta }\right)}^{r_{3}}\\ q=r_{3} \end{array}\right..$$

The equations for parameter estimation based on the POME method can be given as [11]

(12) $$\left\{ \begin{array}{l} -\ln\beta -\frac{1}{r_{3}}\varphi (r_{1})+\frac{1}{r_{3}}\varphi (r_{2})=-E(\ln X)\\ \varphi (r_{2})-\varphi (r_{1}+r_{2})=-E(\ln(1+{\left(\frac{X}{\beta }\right)}^{r_{3}}))\\ \frac{1}{{r_{3}}^{2}}\varphi^{\prime }(r_{1})+\frac{1}{{r_{3}}^{2}}\varphi^{\prime }(r_{2})=\mathrm{var}(\ln X)\\ \varphi^{\prime }(r_{2})-\varphi^{\prime }(r_{1}+r_{2})=\mathrm{var}(\ln(1+{\left(\frac{X}{\beta }\right)}^{r_{3}})) \end{array}\right..$$

### 3.3. Halphen Type A (Hal-A) Distribution

The PDF of the Hal-A distribution is given as

(13a) $$f(x)=\frac{1}{2m^{v}K_{v}(2\alpha )}x^{v-1}\exp[-\alpha (\frac{x}{m}+\frac{m}{x})]\;x>0$$

where *K*_0_(·) is the modified Bessel function of the second kind of order *ν*, *ν* ∈ *R*; and *m* and *α* are parameters, *m* > 0 and *α* > 0.

For deriving Equation (13a) from the entropy theory, the following constraints are specified:

(13b) $$\int_{0}^{\infty }f(x)dx=1$$

(13c) $$\int_{0}^{\infty }f(x)\ln xdx=E(\ln X)$$

(13d) $$\int_{0}^{\infty }xf(x)dx=E(X)$$

(13e) $$\int_{0}^{\infty }\frac{1}{x}f(x)dx=E(\frac{1}{X}).$$

From the entropy theory, the PDF of the Halphen type A distribution can be expressed as [23]

(14) $$f(x)=\exp(-\lambda_{0}-\lambda_{1}\ln x-\lambda_{2}x-\lambda_{3}\frac{1}{x})\;x>0$$

where $\lambda_{3}$ is also the Lagrange multiplier.

The relations between Lagrange multipliers and parameters can be summarized as

(15) $$\left\{ \begin{array}{l} \lambda_{1}=1-v\\ \lambda_{2}=\frac{\alpha }{m}\\ \lambda_{3}=m\alpha \end{array}\right..$$

The equations for parameter estimation based on the POME method can be given as

(16) $$\left\{ \begin{array}{l} \ln m+\frac{1}{K_{v}(2\alpha )}\frac{\partial K_{v}(2\alpha )}{\partial v}=E(\ln X)\\ \frac{mK_{v+1}(2\alpha )}{K_{v}(2\alpha )}=E(X)\\ \frac{K_{v-1}(2\alpha )}{mK_{v}(2\alpha )}=E(\frac{1}{X}) \end{array}\right..$$

### 3.4. Halphen Type B (Hal-B) Distribution

The PDF of the Hal-B distribution can be given as

(17a) $$f(x)=\frac{2}{m^{-2v}ef_{v}(\alpha )}x^{2v-1}\exp[-{\left(\frac{x}{m}\right)}^{2}+\alpha (\frac{m}{x})]\;x>0$$

where $ef_{v}(\cdot )$ is the exponential factorial function, defined as $ef_{v}(\alpha )=2\int_{0}^{\infty }x^{2v-1}\exp[-x^{2}+\alpha x]dx$ (*x* > 0), *m* > 0 are scale parameters, and *v* > 0 and α∈ℜ are shape parameters.

For deriving Equation (17a) from the entropy theory, the following constraints are specified:

(17b) $$\int_{0}^{\infty }f(x)dx=1$$

(17c) $$\int_{0}^{\infty }f(x)\ln xdx=E(\ln X)$$

(17d) $$\int_{0}^{\infty }x^{2}f(x)dx=E(X^{2})$$

(17e) $$\int_{0}^{\infty }xf(x)dx=E(X).$$

From the entropy theory, the PDF of the Halphen type B distribution can be expressed as [23]

(18) $$f(x)=\exp(-\lambda_{0}-\lambda_{1}\ln x-\lambda_{2}x^{2}-\lambda_{3}x)\;x>0.$$

The relations between Lagrange multipliers and parameters can be summarized as

(19) $$\left\{ \begin{array}{l} \lambda_{1}=1-2v\\ \lambda_{2}=\frac{1}{m^{2}}\\ \lambda_{3}=-\frac{\alpha }{m} \end{array}\right..$$

The equations for parameter estimation based on the POME method can be given as

(20) $$\left\{ \begin{array}{l} \ln m+\frac{1}{2ef_{v}(\alpha )}\frac{\partial ef_{v}(\alpha )}{\partial v}=E(\ln X)\\ \frac{m^{2}ef_{v+1}(\alpha )}{ef_{v}(\alpha )}=E(X^{2})\\ \frac{m\cdot ef_{v+\frac{1}{2}}(\alpha )}{ef_{v}(\alpha )}=E(X) \end{array}\right..$$

### 3.5. Halphen Type Inverse B (Hal-IB) Distribution

The PDF of the Hal-IB distribution can be given as

(21a) $$f(x)=\frac{2}{m^{-2v}ef_{v}(\alpha )}x^{-2v-1}\exp[-{\left(\frac{m}{x}\right)}^{2}+\alpha (\frac{m}{x})]\;x>0$$

where *m* > 0 is a scale parameter, and α∈ℜ and *v* > 0 are shape parameters.

For deriving Equation (21a) from the entropy theory, the following constraints are specified:

(21b) $$\int_{0}^{\infty }f(x)dx=1$$

(21c) $$\int_{0}^{\infty }f(x)\ln xdx=E(\ln X)$$

(21d) $$\int_{0}^{\infty }\frac{1}{x^{2}}f(x)dx=E(\frac{1}{X^{2}})$$

(21e) $$\int_{0}^{\infty }\frac{1}{x}f(x)dx=E(\frac{1}{X}).$$

From the entropy theory, the PDF of the Halphen type inverse B can be expressed as [23]

(22) $$f(x)=\exp(-\lambda_{0}-\lambda_{1}\ln x-\lambda_{2}\frac{1}{x^{2}}-\lambda_{3}\frac{1}{x})\;x>0.$$

The relations between Lagrange multipliers and parameters can be summarized as

(23) $$\left\{ \begin{array}{l} \lambda_{1}=2v+1\\ \lambda_{2}=m^{2}\\ \lambda_{3}=-m\alpha \end{array}\right..$$

The equations for parameter estimation based on the POME method can be given as

(24) $$\left\{ \begin{array}{l} \ln m-\frac{1}{2ef_{v}(\alpha )}\frac{\partial ef_{v}(\alpha )}{\partial v}=E(\ln X)\\ \frac{ef_{v+1}(\alpha )}{m^{2}ef_{v}(\alpha )}=E(\frac{1}{X^{2}})\\ \frac{ef_{v+\frac{1}{2}}(\alpha )}{mef_{v}(\alpha )}=E(\frac{1}{X}) \end{array}\right..$$

## 4. Model Selection Based on the Bayesian Technique

First, the five generalized distributions given above were used to fit a given data set **D**, and the equation sets derived by the POME method were applied for estimating their parameters. Second, the Bayesian technique introduced as follows was used to select the most appropriate distribution from the set of distributions for the data set **D**. In this study, the data **D** can be simulated data and observed data. 

Let *I* be the background information. The posterior probabilities over a set of distributions can be expressed as

(25) $$P(M_{i}|D,I)=\frac{P(M_{i}|I)\cdot P(D|M_{i},I)}{P(D|I)}$$

where $P(M_{i}|D,I)$ is the posterior probability of distribution or model $M_{i}$ and indicates the probability of this distribution to be true given the data series **D** and background information *I*. The largest approximate posterior probability among all of the distributions should be chosen as the most appropriate distribution. $P(M_{i}|I)$ is the prior model probability of distribution $M_{i}$; $P(D|M_{i},I)$ is the probabilistic evidence or integrated likelihood of data **D** conditional on model *M_i_*. P(D|I) is a normalization constant and is calculated using the sum and product rules of probability theory as

(26) $$P(D|I)=\sum_{i=1}^{N}P(M_{i}|I)P(D|M_{i},I)$$

where *N* is the number of distributions that are used for the frequency analysis.

To obtain the posterior probability, one needs to calculate the probabilistic evidence $P(D|M_{i},I)$, which can be obtained by integrating a joint distribution $P(\lambda ,D|M_{i},I)$ with respect to vector λ, and can be expressed as

(27) $$P(D|M_{i},I)=\int_{-\infty }^{+\infty }P(\lambda ,D|M_{i},I)d\lambda$$

since

(28) $$P(\lambda ,D|M_{i},I)=P(\lambda |M_{i},I)P(D|\lambda ,M_{i},I)$$

where $P(\lambda |M_{i},I)$ is the prior PDF for the Lagrangian multipliers given distribution *M_i_* and background information *I*. Equation (27) can be obtained as

(29) $$P(D|M_{i},I)=\int_{-\infty }^{+\infty }P(\lambda |M_{i},I)P(D|\lambda ,M_{i},I)d\lambda =E[P(D|\lambda ,M_{i},I)]$$

where $P(D|\lambda ,M_{i},I)$ is the likelihood function of the data in terms of the set of Lagrangian multipliers, and can be expressed by

(30) $$P(D|\lambda ,M_{i},I)=\prod_{k=1}^{n}f(D_{k}|\lambda ,M_{i},I)$$

where *n* is the sample size, and $D_{k}$ denotes a specific value in data set **D**. For a given sample size **D**, model $M_{i}$ and background information *I*, $P(D|\lambda ,M_{i},I)$ can be calculated by the multiplication of all PDF values of *D_k_*.

The multivariate Gaussian distribution was selected as the prior distribution for the Lagrangian multiplier vector λ. The mean value of Lagrangian multipliers was the estimated λ. The covariance matrix Σ was calculated based on the Hessian matrix H, Σ = H^−1^. The equation for calculating the Hessian matrix can be expressed as

(31) $$H=\left[\begin{array}{cccccc} \frac{\partial^{2}\lambda_{0}}{\partial \lambda_{1}^{2}} & \frac{\partial^{2}\lambda_{0}}{\partial \lambda_{1}\partial \lambda_{2}} & . & . & . & \frac{\partial^{2}\lambda_{0}}{\partial \lambda_{1}\partial \lambda_{m}}\\ \frac{\partial^{2}\lambda_{0}}{\partial \lambda_{2}\partial \lambda_{1}} & \frac{\partial^{2}\lambda_{0}}{\partial \lambda_{2}^{2}} & . & . & . & \frac{\partial^{2}\lambda_{0}}{\partial \lambda_{2}\partial \lambda_{m}}\\ . & . & . & . & . & .\\ . & . & . & . & . & .\\ \frac{\partial^{2}\lambda_{0}}{\partial \lambda_{m}\partial \lambda_{1}} & \frac{\partial^{2}\lambda_{0}}{\partial \lambda_{m}\partial \lambda_{2}} & . & . & . & \frac{\partial^{2}\lambda_{0}}{\partial \lambda_{m}^{2}} \end{array}\right].$$

From Equation (29), $P(D|M_{i},I)$ can be obtained by integration. Since the integration in Equation (29) is often a complex and high-dimensional function in Bayesian statistics, the quantity $P(D|M_{i},I)$ was calculated based on the calculation of $E[P(D|\lambda ,M_{i},I)]$.

A Markov Chain Monte Carlo (MCMC) method was used in this study to calculate $P(D|M_{i},I)$ and the posterior distribution of each distribution. The idea of MCMC sampling was first introduced by [24]. Since the target distribution is very complex, we cannot sample from it directly. The indirect method for obtaining samples from the target distribution is to construct an Markov chain with state space *E*, and whose stationary (or invariant) distribution is π(·), as discussed in [25]. Then, if we run the chain for sufficiently long, simulated values from the chain can be treated as a dependent sample from the target distribution. Using the MCMC simulation, pairs of Lagrangian multipliers λ were drawn from the joint distribution $P(\lambda ,D|M_{i},I)$. The quantity $P(D|M_{i},I)$ was finally calculated based on the calculation of $E[P(D|\lambda ,M_{i},I)]$.

**Figure 1:**
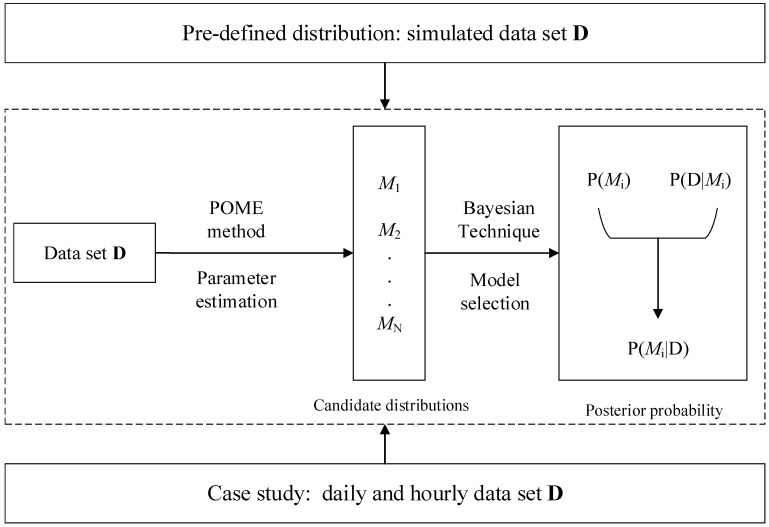
Flowchart of the whole paper. POME: principle of maximum entropy.

In the following, simulated data and real-world data were used for testing the proposed method. The flow chart can be found in Figure 1.

## 5. Performance Evaluation

Before using the proposed method in a practical application, a simulation test was carried out to evaluate the performance of the proposed Bayesian technique for model selection. The simulation test involves the following steps.

First, a distribution with given parameters was pre-defined.

Second, simulated datasets *D* were randomly drawn from the pre-defined distributions.

Third, the Gaussian, lognormal, Gamma, and Weibull distributions were used to fit the data set **D**, and the POME method was applied for parameter estimation.

Fourth, the proposed Bayesian technique was applied for model selection, and the best fitted distributions with the highest posterior probabilities were determined. The results were compared with the pre-defined distributions.

Fifth, the Bayesian model selection technique was compared with commonly used methods in hydrology, such as the root mean square error of the empirical and theoretical probabilities and the AIC criterion.

According to the steps mentioned above, this test focuses on the evaluation of the reliability of the Bayesian model selection for different distributions and data sample sizes. In order to show the performance of the proposed method, some simple and widely used distributions were considered, including the Gaussian, lognormal, Gamma, and Weibull distributions, which involve the Gaussian and non-Gaussian cases. The parameters used for the simulation are given in Table 1. Simulated datasets were randomly drawn from the pre-defined distributions given in Table 1 with sample sizes of 40, 80, 120, 160, 200, and 240. The proposed Bayesian technique was then applied to determine the best fitted distributions for each dataset. The multivariate Gaussian distribution was used for the prior distribution, in which the mean values are the estimated Lagrangian multiplier, and the covariance matrix Σ was calculated based on the Hessian matrix H, Σ = H^−1^. Usually, the estimated parameters were around the true values, so the Gaussian distribution was used. Additionally, the Hessian matrix was calculated to represent the covariance matrix. It is not straightforward to try other distributions, since it is a multivariate problem for which the multivariate Gaussian distribution is widely used.

**Figure 2:**
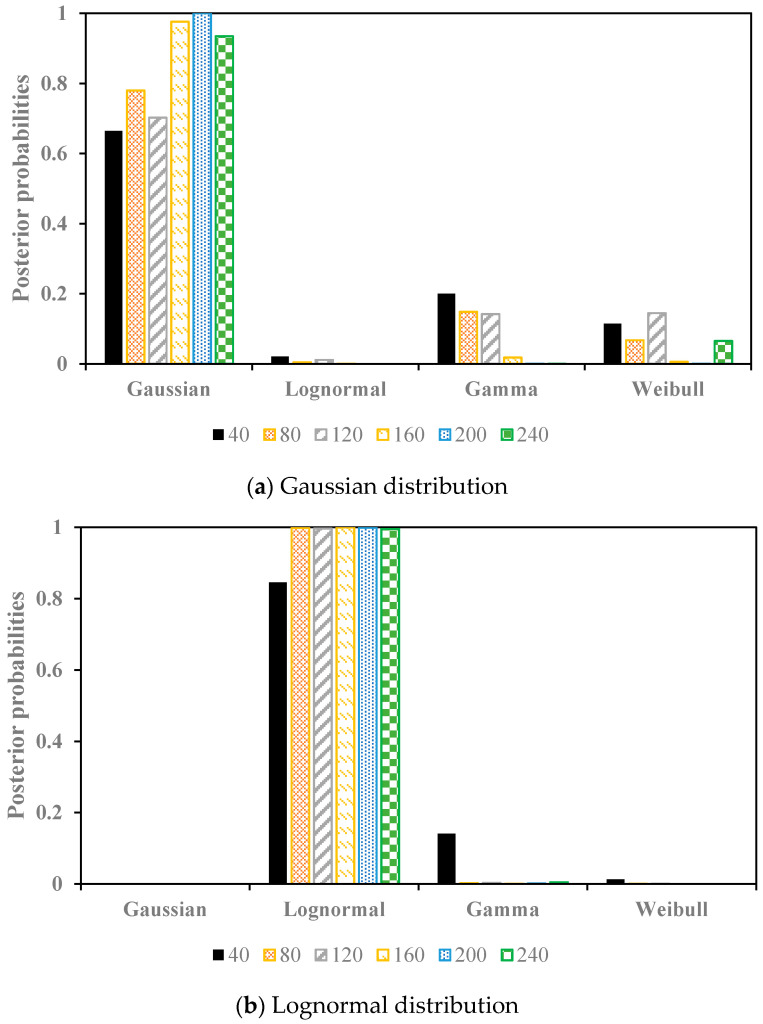

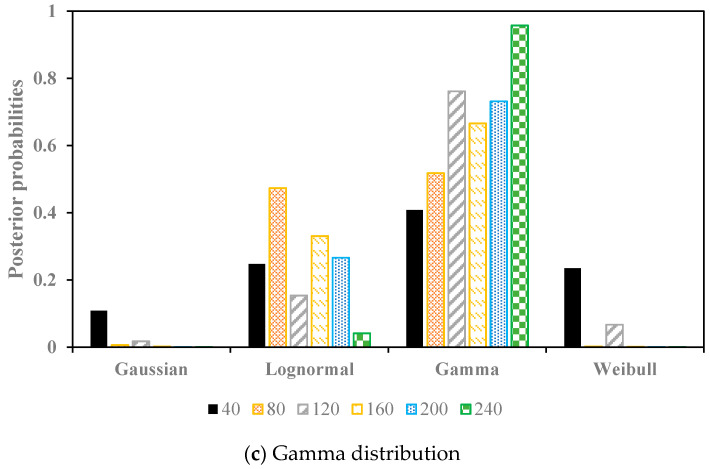
The posterior probabilities of the simulation tests with the Gaussian distribution, Lognormal distribution, and Gamma distribution as the pre-defined distributions, respectively.

The simulation results are shown in Figure 2, which indicate that when the data was sampled from the Gaussian distribution, for all of the sample size, the posterior probabilities of the Gaussian distribution were the highest. For the other tests, namely the lognormal distribution and the gamma distribution as the pre-defined distributions, respectively, the highest posterior probabilities for all of the sample size were the lognormal distribution and gamma distribution as well. Therefore, the proposed Bayesian technique can select the best fitted distribution even for a small sample size (sample size = 40).

The proposed method was compared with the traditional root mean square error (RMSE) and AIC values, which are also used to select the most appropriate distribution. The results are given in Table 2 and Table 3, in which the best fitted distributions with the smallest RMSE and AIC values are in bold. According to the smallest RMSE and AIC values, the correct distribution cannot always be selected. Take the Gaussian distribution as an example. When the sample size was 40, 80, 120, and 160, the best fitted distribution was, respectively, gamma, Weibull, Weibull, and Weibull. When the sample size became larger, greater than 160, the Gaussian distribution was detected as the correct distribution. The RMSE and AIC values of different distributions did not show significantly different results. In other words, the differences in the RMSE and AIC values among those distributions were not large. In Table 3, generally the AIC and RMSE values can show the best fitted distribution. However, in some cases the RMSE and AIC values of different distributions were nearly the same, such as the sample size equaling 160 and 200 in Table 3.

According to the performance test, the Bayesian technique can obtain the correct distribution at any time no matter what the sample size is. On the contrary, the traditional RMSE and AIC do not always work effectively. The RMSE and AIC for the data fitted using different distributions do not shown large differences. Therefore, the proposed method can provide an effective way for model selection in hydrological frequency analysis.

## 6. Case Study

**Figure 3:**
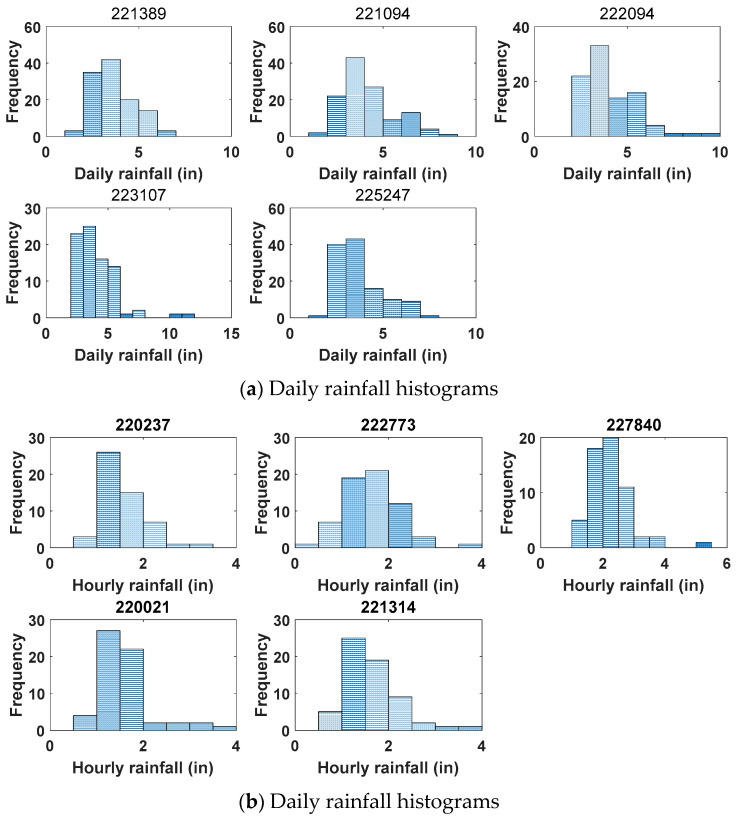
Daily and Hourly rainfall histograms for each gauging station.

Rainfall data for many different timescales were investigated. The timescales of these rainfall dates in the Mississippi River basin ranged from hourly to yearly. The annual maximum daily and hourly series were extracted for frequency analysis, and detailed information of daily and hourly data is shown in Table 4, in which the length of data, the mean value, standard deviation, and the minimum and maximum values are shown. The daily and hourly rainfall histograms for each gauging station are given in Figure 3.

**Figure 4:**
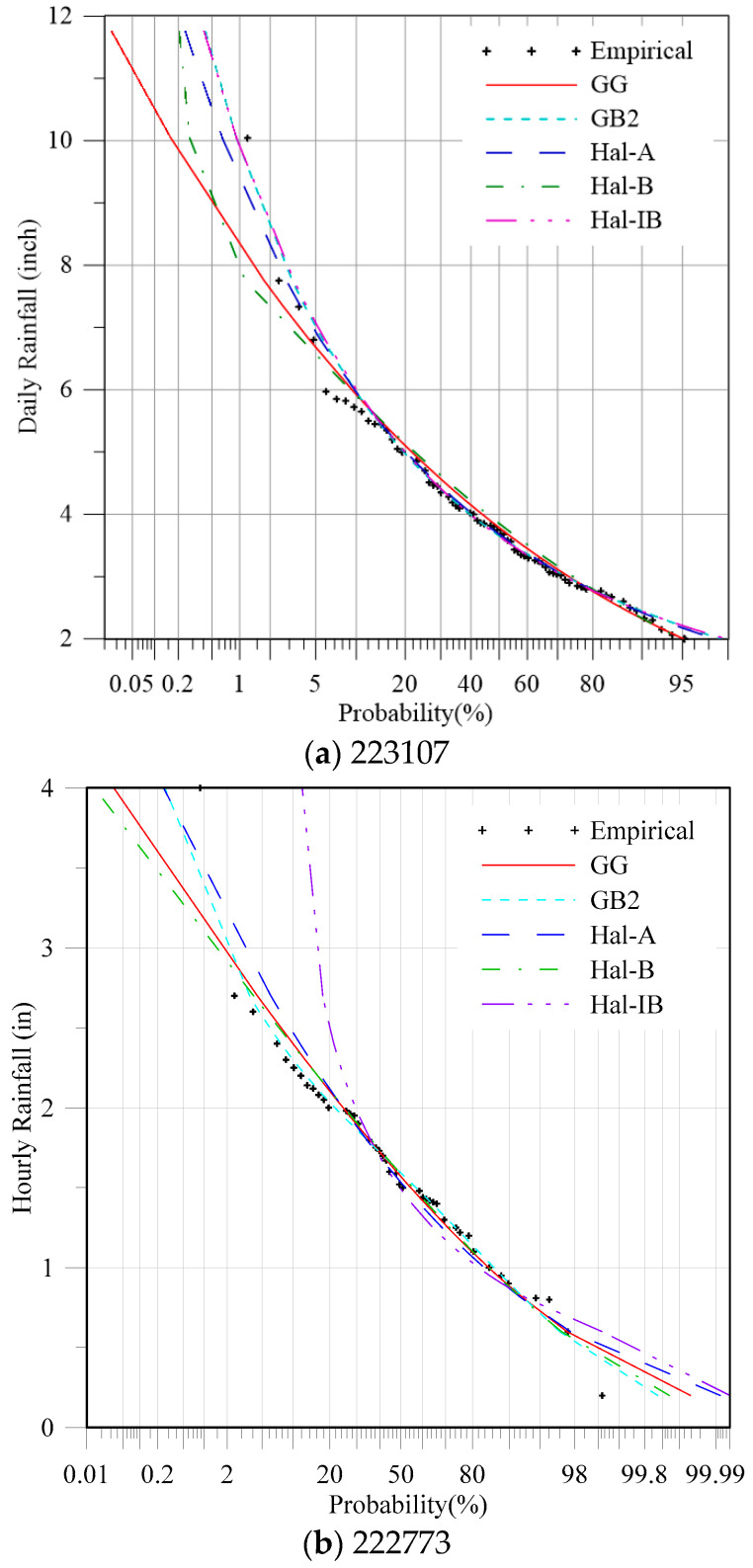
Theoretical and empirical exceedance probabilities of the annual maximum rainfall data at the stations 223107 and 222773.

The five generalized distributions were used to fit the data set, and the entropy method was used to estimate the parameters of these distributions, as given in Table 5 (for daily data) and Table 6 (for hourly data). A full Newton method was used to find the solution of the non-linear equation sets derived before. The “nleqslv” package in R language was used to solve the equation set. The initial value was set as 1 for all potential parameters. The proposed Bayesian technique was used to select the most appropriate distribution for rainfall frequency analysis. The multivariate Gaussian distribution was used for the prior distribution, in which the mean values are the estimated Lagrangian multiplier, and the covariance matrix Σ was calculated based on the Hessian matrix H, Σ = H^−1^. The posterior probabilities are also in Table 5 (for daily data) and Table 6 (for hourly data). The RMSE, AIC, and BIC were also calculated as given in Table 5 and Table 6. Both the AIC and BIC indexes are based on the likelihood values, and a penalty term was introduced for the number of parameters in the model. However, the differences between them are that the penalty term is larger in BIC than in AIC. In this study, it is seen from Table 5 and Table 6 that the selected model by the two methods is the same. Therefore, only the results given by AIC are discussed hereafter. The results indicate that for some of the cases, the selected model based on the three criteria are the same, e.g., gauging stations 225247, 220237, 227840, 220021, and 221314. For some of the stations, the results given by the three methods were not coincident. However, for these cases, the distribution with the lowest AIC value usually had the second-highest posterior probability. Take the gauging station 221094 in Table 5 for example. The AIC and RMSE criteria suggested that the GB2 distribution was the best, for which the posterior probability was 0.34, smaller than the highest one 0.58 (Hal-A). According to the simulation test in Section 4, the performance of the proposed method was better than the traditional AIC and RMSE values. The Bayesian method amplified the differences among the generalized distributions. In order to further compare the performance of these models, the theoretical and empirical exceedance probabilities of the daily rainfall data for the gauging station 223107 are shown in Figure 4a.

According to the results given in Table 5, the best fitted distribution for the gauging station 223107 recommended by the RMSE, AIC, and Bayesian methods, was GB2, Hal-A, and Hal-IB, respectively. As shown in Figure 4a, if the Hal-A distribution was used, the design values for large return periods would be underestimated. The fitting curves of the GB2 and Hal-IB distributions were nearly the same. Thus, the distribution Hal-A recommended by the AIC is not appropriate, and compared with GB2, the Hal-IB with less parameters and higher posterior probability was chosen finally.

The theoretical and empirical exceedance probabilities of the hourly rainfall data for the gauging station 222773 are shown in Figure 4b. According to the results given in Table 6, the best fitted distribution for the gauging station 222773 recommended by the RMSE, AIC and Bayesian methods was GG, Hal-B, and GB2, respectively. As shown in Figure 4b, if the GG and Hal-B distributions were used, the design values for large return periods would be underestimated.

**Figure 5:**
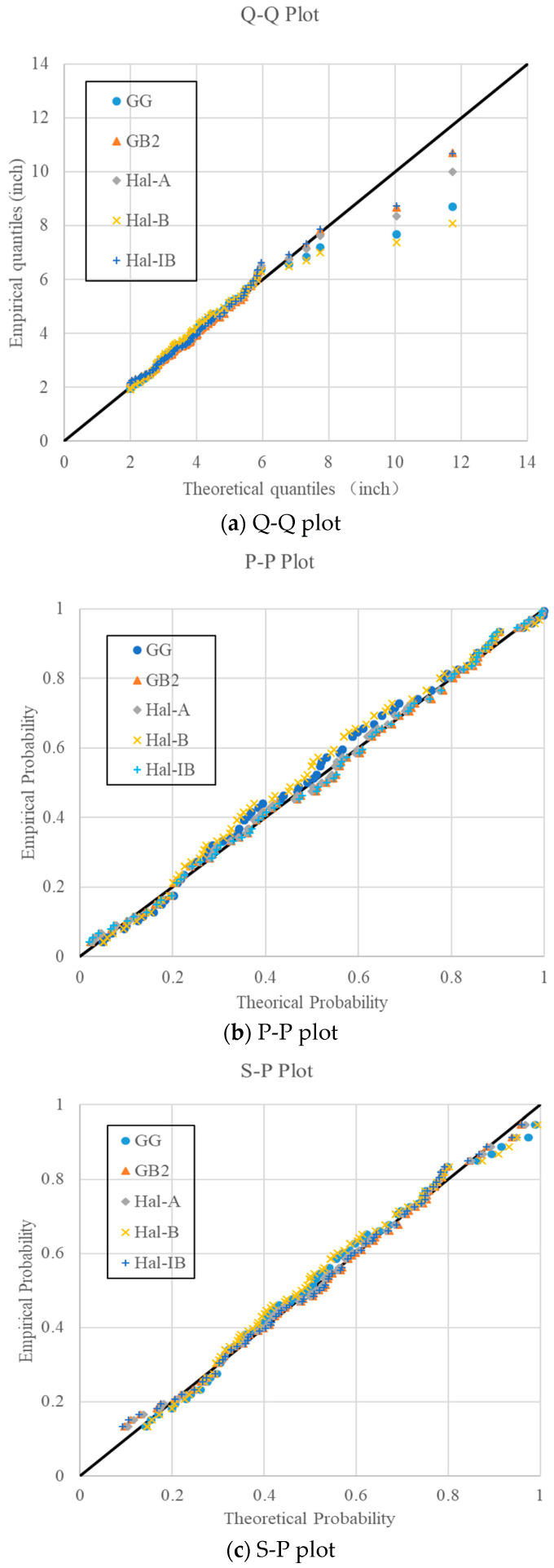
Q-Q, P-P, and S-P plots for the daily rainfall data from the gauging station 223107.

In order to compare the fitting results more comprehensively, the Q-Q plot, P-P plot, and S-P plot were represented for the daily rainfall data from the gauging station 223107 as shown in Figure 5. It can be seen from Figure 5a that the fitting results of GB2 and Hal-IB are nearly the same. When the GG, Hal-A, and Hal-B distributions were used, the design rainfall for a large quantile would be underestimated, since the theoretical rainfall values calculated by the GG, Hal-A, and Hal-B distributions are significantly lower than the observed ones. For the P-P and S-P plots, the differences for large probability are not so obvious, and the plots in Figure 5b,c are well-distributed compared with the Q-Q plot. In Figure 5b, it is easily observed that the Hal-B distribution fits the worst, and the empirical probabilities in the middle part are significantly larger than the theoretical ones. S-P plots remove the impact of variance on the plot, and it is seen that the plots in the S-P figure are much more concentrated than those in the P-P figure.

Furthermore, in the U.S., the Log-Pearson three (LP3) distribution has been recommended for hydrological frequency analysis [26,27]. In order to compare the five generalized distributions with the commonly used LP3 distribution, the six distributions were considered and the proposed Bayesian method was used to select the best fitted one. The results are given in Table 7.

## 7. Conclusions and Discussion

The paper proposes a model selection approach based on a Bayesian technique to choose the best fitted distribution for hydrological frequency analysis. Five generalized distributions, including GG, GB2, Hal-A, Hal-B, and Hal-IB, which are also widely used in hydrology, were considered. The entropy-based method was used to express these distributions and the POME method was applied for parameter estimation. A simulation test was carried out to evaluate the performance of the proposed Bayesian method. Daily rainfall data from five stations and hourly rainfall data from another five stations from the Mississippi basin were selected as case studies. The main conclusions are summarized as follows.

(1) The entropy-based five generalized distributions are given, and their corresponding equation sets for parameter estimation are introduced. The results of simulation test and case study show that the POME method can provide an effective way for parameter estimation.
(2) Results of the simulation test demonstrate that the Bayesian technique can choose the most suitable distribution. Compared with the commonly used RMSE and AIC values, the proposed method gives a better performance.
(3) Results of the case study indicate that when using different criteria for model selection, the results are not always the same. For some of the cases, the three criteria choose the same distribution. For others, the results are slightly different. Since choosing the probable distribution for hydraulic design is very significant, especially for extreme magnitudes, the distribution should be selected carefully. According to the posterior probabilities calculated by the proposed method for daily and hourly data from 10 gauging stations, generally the Hal-IB distributions give better fits for daily data and GB2 distributions give better fits for hourly data.
(4) According to the results of the simulation test and case studies, the Bayesian model selection technique can give a more reliable result than the traditional RMSE and AIC values. Thus, the proposed method provides an effective way for model selection for hydrological frequency analysis.
(5) The significant contribution of this paper is that compared with the traditional method, the proposed method is based on entropy theory, and the posterior probabilities were calculated based on the generation of Lagrange multipliers. In addition, the five generalized distributions were involved in this paper, since previous research mainly focus on the commonly used distribution or standard distributions.

This contribution of this paper mainly concentrates on univariate hydrometeorological frequency analysis. Recently, multivariate hydrological analysis has also surged up, such as [2,4,28,29,30,31]. However, univariate frequency analysis is the basis of multivariate frequency analysis, which can provide the marginal distributions for joint distribution. Thus, before establishing the multivariate distributions, the univariate distribution should be built rationally and appropriately first.

In addition, in the common hydrological frequency analysis, the hydrological data set is assumed to be independent and identically distributed [1]. Since there are influences of climate change and human activities on streamflow, it is possible that the mean value or the variation of the whole series would be changed. In other words, the data set is non-stationary. Non-stationary hydrological frequency analysis is also another hot and difficult topic in hydrology recently. In this paper, we mainly focus on the stationary frequency analyses of hydrometeorological extremes. Non-stationary hydrological frequency analysis will be discussed in future research.

Although this paper discussed the model selection method based on the five generalized distributions, the traditional commonly used distribution, the LP3 distribution, is still an effective tool for frequency analysis and can be used for design rainfall or flood calculation.

## Figures and Tables

**Table 1:** Parameters of different distributions for simulation test.

Number	Distribution	Probability Density Function (PDF)	Parameters
1	Gaussian	$f(x)=\frac{1}{\sqrt{2\pi }\sigma }\exp(-\frac{{\left(x-\mu \right)}^{2}}{2\sigma^{2}})$	$\begin{array}{l} \mu =10,\\ \sigma =3.162 \end{array}$
2	Lognormal	$f(x;\mu ,\sigma )=\left\{ \begin{array}{l} \frac{1}{\sqrt{2\pi }\sigma x}\exp[-\frac{1}{2\sigma^{2}}{\left(\ln x-\mu \right)}^{2}],\;x>0\\ 0,\;x\leq 0 \end{array}\right.$	$\begin{array}{l} \mu =2,\\ \sigma =0.6 \end{array}$
3	Gamma	$f(x;\;\beta ,\alpha )=\frac{\beta^{\alpha }}{\Gamma (\alpha )}x^{\alpha -1}e^{-\beta x},\;x>0$	$\begin{array}{l} \alpha =10,\\ \beta =1 \end{array}$

**Table 2:** The root mean square error (RMSE) and Akaike Information Criterion (AIC) values for the simulation test, in which the pre-defined distribution is the Gaussian distribution.

Distributions	Criteria	40	80	120	160	200	240
Gaussian	RMSE	0.03	0.0175	0.0247	0.0243	**0.0118**	**0.0115**
	AIC	−169.07	−443.78	−577.56	−792.76	**−1244.68**	**−1482.07**
lognormal	RMSE	0.0385	0.044	0.025	0.05	0.057	0.0437
	AIC	−155.12	−279.77	−558.39	−525.84	−683.75	−913.47
Gamma	RMSE	**0.0239**	0.0293	0.0161	0.0377	0.0367	0.026
	AIC	**−177.05**	−332.5793	−642.25	−607.66	−801.37	−1116.59
Weibull	RMSE	0.03	**0.0168**	**0.016**	**0.0168**	0.017	0.0118
	AIC	−167.34	**−441.3**	**−661.23**	**−858.42**	−1082.21	−1457.45

**Table 3:** The RMSE and AIC values for the simulation test, in which the pre-defined distribution is the Gamma distribution.

Distributions	Criteria	40	80	120	160	200	240
Gaussian	RMSE	0.0409	0.0317	0.33	0.0333	0.0318	0.033
	AIC	−152.98	−372.77	−509.91	−693.5	−879.39	−1030.29
lognormal	RMSE	0.0217	0.0161	0.0211	**0.0098**	0.0158	0.0179
	AIC	−185.92	−437.1	−582.86	−1018.23	**−1094.31**	−1311.04
Gamma	RMSE	**0.0229**	**0.0141**	**0.0169**	0.0108	**0.0124**	0.0126
	AIC	**−189.06**	**−486.4**	**−632.77**	**−1026.04**	−1191.77	**−1421.8**
Weibull	RMSE	0.042	0.0398	0.0314	0.036	0.0344	0.0332
	AIC	−150.55	−373.95	−523.74	−672.99	−853.67	−1053.41

**Table 4:** Detailed information of daily and hourly annual maximum rainfall series.

Times	Gauging Station	Number	Length of Data	Mean Values	SD	Max	Min
Daily	Canton gauging	221389	1893–2012	3.6	1.1	6.8	1.65
	Brookhaven City, MS	221094	1894–2014	4.14	1.44	8.08	1.85
	Crvstal Spgs Exp Stn, MS	222094	1893–1954, 1985–2014	4	1.4	9.04	2.02
	Forest, MS	223107	1930–2012	4.02	1.66	11.75	2
	Louisville, MS	225247	1895–2014	3.65	1.28	7.47	1.7
Hourly	Arkabutla dam	220237	1949–2001	1.57	0.46	3.12	0.88
	Enid dam MS	222773	1949–2012	1.62	0.59	4	0.2
	Saucier experimental forest MS	227840	1955–2013	2.23	0.7	5.13	1.2
	Aberdeen MS	220021	1952–2011	1.55	0.62	3.8	0.7
	Calhoun city MS	221314	1948–2009	1.6	0.57	3.88	0.8

**Table 5:** Parameters, RMSE, AIC, and posterior probabilities for daily data calculated by five generalized distributions.

Number	Distribution	Para1	Para2	Para3	Para4	RMSE	AIC	BIC	Posterior Probabilities
221389	GG	13.251	0.8397	0.1335		0.0205	−613.05	−604.76	0.02
	Gb2	0.9955	1.5808	34.95	16.84	0.0195	−605.68	−597.39	0.08
	Hal-A	5.235	3.431	0.0327		**0.0183**	**−624.56**	**−616.27**	0.14
	Hal-B	−20.927	7.363	5.381		0.0237	−593.21	−584.93	0.02
	Hal-IB	−9.103	2.855	4.776		0.0233	−544.21	−535.92	**0.74**
221094	GG	13.308	0.684	0.0527		0.0362	−482.72	−477.32	0.08
	Gb2	1.678	2.203	11.994	4.894	**0.0203**	**−607.37**	**−601.98**	0.34
	Hal-A	3.507	7.812	−5.534		0.0229	−584.34	−578.95	**0.58**
	Hal-B	−20.918	8.0322	5.6835		0.0557	−409.26	−410.15	0.00
	Hal-IB	−10.755	2.319	3.699		0.0525	−539.94	−531.55	0.00
222094	GG	14.855	0.628	0.0255		0.0307	−412.5	−404.94	0.04
	Gb2	1.3803	1.38	24.977	6.573	0.0213	−462.61	−455.04	0.20
	Hal-A	2.5669	12.3193	−7.9616		**0.021**	**−466.21**	**−458.64**	0.32
	Hal-B	−12.2589	7.581	3.5971		0.04	−385.6	−378.04	0.01
	Hal-IB	−9.546	2.2708	4.2153		0.0218	−464.18	−456.61	**0.44**
223107	GG	13.5999	0.5606	0.0131		0.0294	−409.36	−402.11	0.00
	Gb2	2.199	1.837	9.931	2.4389	**0.0164**	−466.07	−458.81	0.08
	Hal-A	0.977	30.104	−8.194		0.0168	**−479.18**	**−471.93**	0.21
	Hal-B	−27.5605	14.5778	3.8889		0.0406	−362.58	−355.33	0.00
	Hal-IB	−3.8918	3.7076	3.2608		0.0165	−457.91	−450.65	**0.71**
225247	GG	13.596	0.6657	0.0384		0.0437	−433.23	−424.86	0.00
	Gb2	1.822	1.156	23.62	3.641	0.02456	−565.73	−557.36	0.00
	Hal-A	2.0554	14.775	−8.756		0.0287	−533.57	−525.21	0.04
	Hal-B	−46.515	14.128	6.0834		0.0653	−363.04	−354.68	0.00
	Hal-IB	−3.361	3.938	3.602		**0.0232**	**−575.37**	**−567.01**	**0.96**

**Table 6:** Parameters, RMSE, AIC, and posterior probabilities for hourly data calculated by five generalized distributions.

Number	Distribution	Para1	Para2	Para3	Para4	RMSE	AIC	BIC	Posterior Probabilities
220237	GG	−19.179	0.6794	0.0284		0.048	−187.74	−181.83	0.20
	GB2	2.963	2.5391	6.8892	2.4497	**0.0308**	**−233.74**	**−227.83**	**0.57**
	Hal-A	3.0259	15.972	−12.521		0.0344	−215.46	−209.55	0.08
	Hal-B	−27.458	8.384	6.6655		0.0523	−178.6	−172.69	0.10
	Hal-IB	−7.185	4.2898	5.7116		0.0312	−222.62	−216.72	0.06
222773	GG	4.169	1.858	1.656		**0.0318**	−291.31	−284.83	0.13
	GB2	4.5	3.221	0.775	2.153	0.0337	−291.92	−285.44	**0.70**
	Hal-A	5.36 × 10^−2^	1.90 × 10^−2^	6.8204		0.0333	−274.18	−267.70	0.05
	Hal-B	1.3105	1.6132	1.5702		0.0338	**−292.69**	**−286.21**	0.12
	Hal-IB	−50.5719	0.1565	3.0044		0.0419	−231.43	−224.95	0.00
227840	GG	17.0415	0.6977	0.0225		0.0473	−266.6	−260.37	0.05
	GB2	2.013	1.2966	11.7277	4.6042	**0.0266**	**−275.55**	−266.32	**0.84**
	Hal-A	2.7995	8.6585	−11.0919		0.0271	−274.68	**−268.45**	0.00
	Hal-B	−23.6777	4.7218	5.8388		0.0422	−249.55	−243.32	0.11
	Hal-IB	−10.601	1.865	5.7014		0.0316	−264.11	−257.88	0.00
220021	GG	11.875	0.65	0.0173		0.0582	−189.32	−183.04	0.10
	GB2	4.1081	0.9324	3.7284	0.9621	0.0468	−221.54	−212.26	0.33
	Hal-A	1.1704	10.6076	−8.8785		0.0532	−217.75	−211.47	0.15
	Hal-B	−30.3037	5.6947	4.203		0.0761	−181.74	−175.42	0.00
	Hal-IB	0.7828	2.248	2.3074		**0.0455**	**−228.4**	**−222.12**	**0.42**
221314	GG	14.1465	0.6738	0.0171		0.0405	−281.77	−275.39	0.00
	GB2	1.5734	0.3406	44.9999	4.516	0.0318	−293.19	−286.81	0.05
	Hal-A	1.7786	7.979	−9.475		0.0266	−316.06	**−309.68**	0.11
	Hal-B	−27.792	4.9387	4.622		0.0432	−263.62	−257.24	0.00
	Hal-IB	−6.217	1.438	4.185		**0.0249**	**−309.73**	−303.35	**0.84**

**Table 7:** Parameters, RMSE, AIC, and poster probabilities for 223107 daily data calculated by five generalized distributions and the Log-Pearson three (LP3) distribution.

Distributions	Para1	Para2	Para3	Para4	RMSE	AIC	BIC	Posterior Probabilities
GG	13.60	0.56	0.013		0.0294	−406.36	−402.11	0.002
GB2	2.20	1.84	9.93	2.44	**0.0164**	−463.07	−460.81	0.071
Hal-A	0.98	30.10	−8.19		0.0168	**−476.18**	**−471.93**	0.197
Hal-B	−27.56	14.58	3.89		0.0406	−359.58	−355.33	0.0003
Hal-IB	−3.89	3.70	3.26		0.0165	−454.91	−450.77	**0.674**
LP3	14.29	0.09	−0.03		0.0167	−448.65	−444.39	0.056
